# Volvulus total du grêle sur mésentère commun incomplet, une complication exceptionnelle chez l’adulte: à propos d’un cas

**DOI:** 10.11604/pamj.2019.33.220.18159

**Published:** 2019-07-17

**Authors:** Aliou Zabeirou Oudou, Ismael Dandakoye Soumana, Tarek Souiki, Karim Ibn Majdoub, Imane Toughrai, Said Ait Laalim, Khalid Mazaz

**Affiliations:** 1 Service de Chirurgie Viscérale B, CHU Hassan 2, Fes, Maroc

**Keywords:** Volvulus total, intestin grêle, mésentère commun incomplet, anomalie de rotation, Total volvulus, small intestine, common incomplete mesentery, abnormal rotation

## Abstract

Le volvulus total du grêle complique le mésentère commun incomplet correspond à un arrêt de la rotation à 180° de l'anse intestinale primitive. La racine du mésentère est très courte et l'ensemble de l'intestin grêle se trouve pédiculé sur l'axe artériel mésentérique supérieur. Cette situation est à très haut risque de volvulus du grêle et d'infarctus entero-mésentérique. Le volvulus aigu impose une intervention chirurgicale en urgence; l'imagerie ne doit pas retarder la prise en charge chirurgicale. Le geste chirurgical consiste à la détorsion du volvulus (dans le sens antihoraire), la viabilité de l'intestin appréciée. L'intestin est rangé position de mésentère commun complet: cœcum dans la fosse iliaque gauche. Nous rapportons l'observation d'un patient de 60 ans admis pour volvulus total du grêle sur mésentère commun incomplet, opéré en urgence avec une évolution postopératoire favorable.

## Abstract (en)

Total small bowel volvulus complicating common incomplete mesentery is an arrest of rotation of the primary intestinal loop at 180°. The root of the mesentery is very short and the whole small intestine is located on the superior mesenteric artery axis. Patients are at very high risk of small bowel volvulus and enteromesenteric infarction. Acute volvulus requires emergency surgery; imaging must not delay surgery. Surgery is based on the untwisting of the volvulus (counterclockwise) after the assessment of intestinal viability. The intestine placed in the complete common mesentery position: the cœcum is situated in the right iliac region. We report the case of a 60-year old patient admitted with total small bowel volvulus on an incomplete common mesentery who underwent emergency surgery with favorable postoperative outcome.

## Introduction

Le volvulus total du grêle sur anomalie de rotation de l'anse intestinale primitive est exceptionnel chez l'adulte puisqu'une centaine de cas seulement ont été rapportés [1]. Le mésentère commun résulte d'une anomalie de rotation du tube digestif. Il est caractérisé par la persistance d'une disposition anatomique embryonnaire secondaire à une anomalie de rotation de l'anse ombilicale primitive, constituant ainsi un méso commun à toute l'anse intestinale et une racine du mésentère extrêmement courte. Cette insuffisance de rotation est le plus souvent associée à un défaut d'accolement [2]. Ces anomalies de rotation intestinale peuvent aboutir à des complications redoutables parfois mortelles, qui surviennent généralement au cours de la période néonatale où à l'âge pédiatrique. On estime que la prévalence de ces malformations congénitales à l'âge adulte est de l'ordre de 0,2% à 0,5%; âge auquel elles demeurent très souvent asymptomatiques et donc non diagnostiquées [3]. Le diagnostic de volvulus total du grêle peut se faire dans des circonstances très variées: en urgence devant un tableau d'occlusion intestinale aiguë, voire un état de choc pouvant conduire au décès, devant un tableau de douleurs abdominales répétées plus ou moins associées à des troubles du transit. Nous rapportons l'observation d'un patient de 60 ans admis pour volvulus total du grêle sur mésentère commun incomplet, opéré en urgence avec une évolution postopératoire favorable.

## Patient et observation

**Figure 1:**
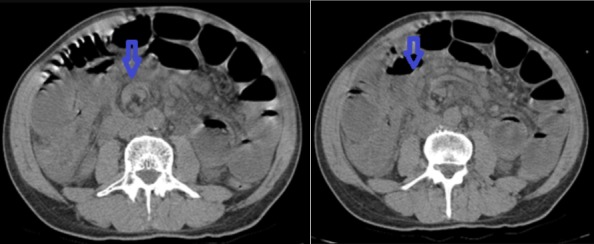
TDM coupe axiale montrant l’image en tourbillon

**Figure 2:**
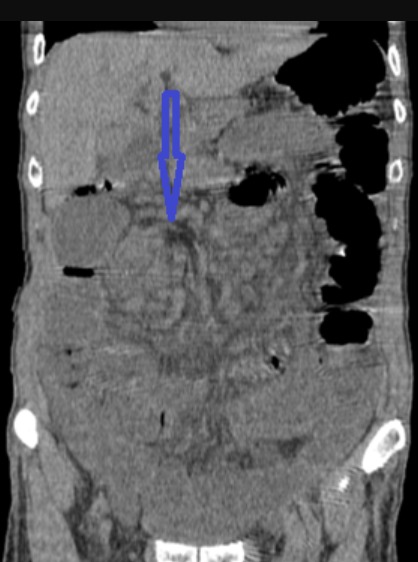
TDM, coupe coronale montrant l’image en tourbillon

**Figure 3:**
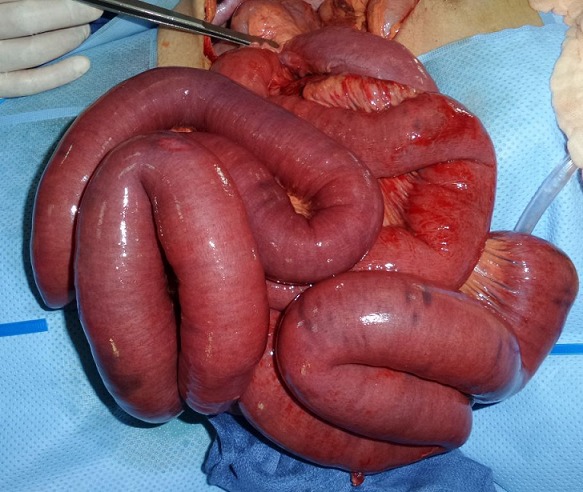
image per-opératoire montrant le volvulus avec souffrance intestinale

**Figure 4:**
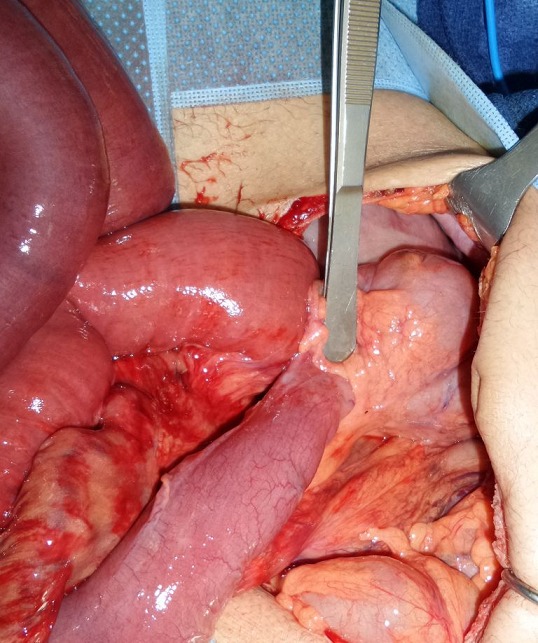
image per-opératoire montrant le cœcum en sous hépatique

**Figure 5:**
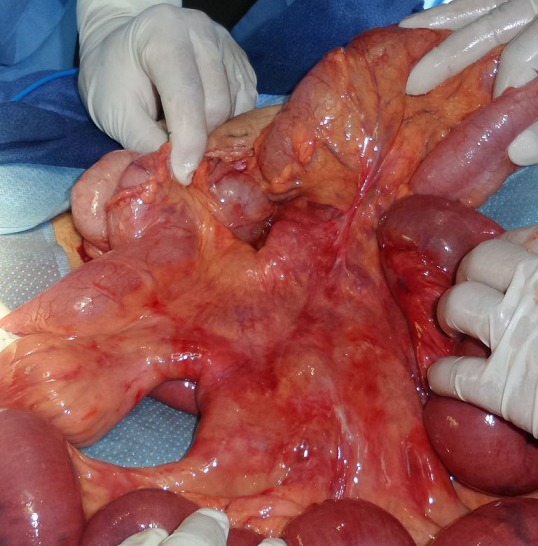
image per-opératoire montrant le mésentère commun incomplet

Il s'agit d'un patient âgé de 60 ans sans antécédent pathologique connu admis dans notre formation dans un tableau de syndrome occlusif, associé à des douleurs abdominales intenses et insupportables et des vomissements. Cette symptomatologie évoluait depuis 2 heures de temps avant son arrivée au service des urgences. L'examen à son admission trouvait un patient: conscient GCS à 15, déshydraté avec une pression artérielle à 110mmHg/70mmHg; une fréquence cardiaque à 100 battements/min; polypnée à 30 cycles/minutes; apyrétique à 37°C. Par ailleurs l'examen abdominal trouvait un abdomen distendu, tympanique à la percussion sans contracture ni défense. Un bilan biologique réalisé en urgence a mis en évidence une insuffisance rénale d'allure fonctionnelle, une hypokaliémie et une hyponatrémie. Pas de syndrome inflammatoire biologique. Un abdomen sans préparation (ASP) a été réalisé montrant des niveaux hydroaériques type grélique. Nous avons complété par une tomodensitométrie (TDM) abdomino-pelvienne non injectée (vue insuffisance rénale) qui trouvait une image en tourbillon intéressant les anses jéjunales, avec le cœcum en position sous hépatique et les anses grélique à droite (Figure 1, Figure 2). Le diagnostic d'occlusion sur mésentère commun incomplet a été retenu. Après une courte réanimation, le patient fut admis d'urgence au bloc opératoire. L'exploration trouvait tout le grêle distendu et souffrant avec une tour de spire concernant la première anse jéjunale et la dernière anse iléale (Figure 3). Le cœcum se trouve en sous hépatique accolé à la paroi par une bride de Ladd (Figure 4). Le geste chirurgical a consisté à une détorsion dans le sens antihoraire suivi d'une recoloration immédiate de l'intestin grêle, puis cure de l'anomalie embryologique de rotation selon la procédure de Ladd (section des brides, transformation du mésentère commun incomplet en mésentère commun complet pour éviter toute récidive et enfin d'une appendicectomie de principe (Figure 5)). L'évolution a été favorable le patient est sortie après 4 jours d'hospitalisation.

## Discussion

L'interruption de la rotation intestinale à 180° aboutit à une position où la jonction iléocæcale vient se fixer dans la région sous-hépatique [1-6]. Cet accolement, s'il est situé en regard du duodénum, peut inconstamment provoquer une compression extrinsèque du premier ou du deuxième duodénum: on parle alors de « brides de Ladd ». L'angle duodénojéjunal se situe, quant à lui, à droite du rachis. La première anse jéjunale et la dernière anse iléale se trouvent donc à proximité de l'axe mésentérique supérieur et très proche l'une de l'autre. Parfois, il peut même exister un accolement congénital entre le méso de ces deux anses intestinales (« fusion mésentérique de Pellerin » [7]). Dans cette position à 180°, la racine du mésentère est extrêmement courte et l'ensemble de l'intestin grêle se trouve « pédiculé » sur son axe vasculaire mésentérique supérieur. Cette position, dite en « mésentère commun incomplet », est à haut risque de Volvulus total du grêle du fait de la brièveté de la racine du mésentère et de son absence d'accolement.

Le diagnostic de volvulus total du grêle peut se faire dans des circonstances très variées. En urgence, devant un tableau d'occlusion intestinale aiguë, voire un état de choc pouvant conduire au décès. Devant un tableau de douleurs abdominales répétées plus ou moins associées à des troubles de transit [8]; plus rarement, au décours d'une chirurgie laparoscopique, comme cela a été décrit après une cholécystectomie [9], une appendicectomie [10] ou une chirurgie de l'obésité [11]. Le volvulus total du grêle sur mésentère commun incomplet ayant une symptomatologie peu spécifique, il est essentiel de penser à évoquer précocement ce diagnostic, afin d'être en mesure de le confirmer, idéalement en préopératoire, par un examen tomodensitométrique. La tomodensitométrie avec injection de produit de contraste est l'examen de référence pour le diagnostic de volvulus total du grêle sur mésentère commun incomplet chez l'adulte [12]. Décrit la première fois par Fisher en 1981 sous le nom de whirl-like pattern, le signe du « tourbillon » semble en effet être pathognomonique du volvulus total du grêle pour la majorité des auteurs [13]. Il correspond à la vrille du mésentère visible en position médiane, en avant de l'aorte et au niveau de l'artère mésentérique supérieure, autour de laquelle viennent « s'enrouler » la veine mésentérique supérieure et le jéjunum proximal. Les clichés avec injections permettent de visualiser la verticalisation, ou l'inversion, des vaisseaux mésentériques supérieurs, avec une veine se plaçant au-dessus ou à gauche de l'artère [14], bien que ce signe ne soit pas constant. L'épaisseur de cette masse de tourbillon serait Proportionnelle au degré de rotation du volvulus, mais il est plus précis d'évaluer le degré de rotation en calculant le nombre de spires réalisés par les vaisseaux mésentériques [15].

La connaissance de l'anatomie du mésentère commun incomplet est indispensable pour en faire le diagnostic en peropératoire et comprendre les principes de sa cure chirurgicale. Dans la forme typique de la rotation intestinale à 180° dite en mésentère commun incomplet: le duodénum est court s'interrompant après D2 avec un angle de Treitz situé à droite du rachis; un cæcum en position sous-hépatique; une racine du mésentère très courte, centrée par l'axe vasculairemésentérique supérieur et donnant le plus souvent un aspect pédiculé du mésentère [16]. Devant un tableau d'occlusion aigue, la laparotomie médiane doit être choisie de première intention. Le volvulus total du grêle est identifié sur le fait que l'ensemble du grêle est intéressé par le volvulus et se trouve, une fois extériorisé, pédiculisé sur son mésentère. L'inspection du mésentère révèle alors la présence d'un ou plusieurs tours de spires. À ce stade, il est important de noter le sens du volvulus (le plus souvent horaire), le nombre approximatif de tours de spires et la coloration du grêle [17]. Le mésentère commun incomplet est identifié par la position non anatomique du cæcum (et ses éventuelles adhérences en regard du duodénum), la position de l'angle de Treitz à droite du rachis et le défaut d'accolement du mésentère dont la racine apparaît toujours très courte [18]. La procédure de Ladd reste à ce jour le traitement de référence du volvulus total du grêle (VTG) sur anomalie de rotation (AR) aussi bien chez l'adulte que chez l'enfant. Cette procédure consiste en une réduction du volvulus, suivie d'une mise en mésentère commun complet de l'intestin grêle pour éviter toute récidive du volvulus. Vient ensuite le temps d'une appendicectomie de principe. L'appendice doit en effet être systématiquement retiré afin que le patient ne coure pas le risque de faire ultérieurement un épisode d'appendicite aiguë en position ectopique. Positionnement en mésentère commun complet [19, 20].

## Conclusion

Le volvulus total du grêle sur mésentère commun incomplet ayant une symptomatologie peu spécifique, il est essentiel de penser à évoquer précocement ce diagnostic, afin d'être en mesure de le confirmer, idéalement en préopératoire, par un examen tomodensitométrique avec injection. À défaut, tout chirurgien d'adultes doit au moins savoir diagnostiquer à ventre ouvert. Le volvulus total du grêle sur mésentère commun incomplet et sa complication et connaître les principes de son traitement, selon la procédure de Ladd.

## Conflits d’intérêts

Les auteurs ne déclarent aucun conflit d'intérêts.
